# Suture of War Wounds1.This paper was first given as a lecture in French at Bouleuse, Jan. 17, 1918. It was first given in English before the Research Society of the American Red Cross in France, in February 1918, and was first published in *The Medical Bulletin* for March 1918, vol. 1, p. 292. It is reprinted in this number of *War Medicine* by special request of the Field Service School for American Medical Officers. It has been revised by Doctor Lemaître, at whose request the additional note by Doctor René Bloch is appended.

**Published:** 1918-12

**Authors:** René Lemaitre


### SUTURE OF WAR WOUNDS[1]

By Doctor RENÉ LEMAITRE

### PART FIRST

I have practised primary suture of war wounds since the month of July, 1915. After 31 months' experience it seems interesting to publish the results I have obtained, the reasons that led me to treat war wounds in this fashion, and the technique I have used. I had the advantage of working in an army ambulance immobilized in a large hospital center, the inactivity of which in a majority of cases allowed me to keep the wounded until recovery; and later on in a mere active center, with a capacity allowing me to keep a patient for a prolonged period. I can therefore give these results as definite for the majority of the cases I have treated. Almost all, in fact, outside of fracture cases, joined their regiment directly after leaving the hospital, or within a period of from 15 days to two months after operation. They were then given a leave of absence varying from 15 days to three months. During this time 2381 wounded were treated within 24 hours after their injury : the immense majority, 1951, between the 7th hour and the 15th, 302 before the 7th hour, and 123 after the 15th following injury.

We believe it to be preferable in any large number of cases, and especially in those with multiple wounds, to speak of wounds rather than of wounded. Each wound can be considered from the point of view of treatment and evolution as though it were unique. We shall, therefore, speak of wounds and not of wounded. These 2301 wounded presented 4306 wounds. Amongst these wounds 334 were not treated surgically on account of their small size (very small fragments, especially in the face, perforating rifle bullet wounds with entrance and exit-openings equally small, without fracture, without injury to the blood vessels or the nerves and without any sign of infection). — 4072 *wounds were*

1. This paper was first given as a lecture in French at Bouleuse, Jan. 17, 1918. It was first given in English before the Research Society of the American Red Cross in France, in February 1918, and was first published in *The Medical Bulletin* for March 1918, vol. 1, p. 292. It is reprinted in this number of *War Medicine* by special request of the Field Service School for American Medical Officers. It has been revised by Doctor Lemaitre, at whose request the additional note by Doctor René Bloch is appended.

*treated surgically*, 2664 of these were *united by primary suture*, 231 *by delayed primary suture*, and 324 by *secondary suture*. 833 wounds were not sutured at all, either because the wounds closed progressively of themselves, because the wounded died, or, finally, because in a period of great activity the wounded had to be evacuated before they could be sutured. In our statistics we will consider only the 2664 primary sutures and omit mention of the 231 delayed primary sutures and of the 324 secondary sutures; also of the 853 wounds which were not sutured at all[1].

*Wounds of the Soft Parts.*

1. Wounds of the soft parts of medium importance . . . . . . 770
   with 3 complete failures and 9 partial failures[2].
2. Wounds of the soft parts of greater importance. . . . . . 1107
   with 14 complete failures and 19 incomplete failures.
3. Wounds of the bone without complete fracture . . . . . . 159
   with 5 complete failures and 4 partial ones.
4. Wounds of the soft parts with injury to a large blood vessel. 68
   with 2 complete failures and 5 partial ones.
5. Wounds of the soft parts with injury to a large nerve trunk. 34
   with no complete failure and 4 partial ones.

*Wounds of the Large Joints.*

1. Wounds of the tibio-tarsal (with 4 cases of astragalectomy). 15
   with no complete failure and 2 partial ones.
2. Wounds of the knee (with 3 immediate resections of the knee joint) . . . . . . . . . . . . . . . . . . . . . 22
   with one complete failure (cured after secondary resection).
3. Wounds of the wrist (of which 3 presented limited bone lesions of the wrist necessitating partial excision of the carpus) . . . . . . . . . . . . . . . . . . . . . . . 20
   with no complete failure and 2 partial ones.
4. Wounds of the elbow joint (of which 12 underwent partial resection . . . . . . . . . . . . . . . . . . . . . . 22
   with no complete failure and 3 partial ones.
5. Wounds of the shoulder joint (of which 2 were treated by immediate resection of the head of the humerus). . . . 12
   with no complete failure and 1 partial one.

1. See Additional Note by Doctor René Bloch, p. 827.
2. We understand by " complete failures " cases where it was necessary to remove all the stitches; and by " partial failures " those where sooner or later after the operation we had to remove a few stitches, the rest remaining in place, the infection being superficial and easily eliminated.

*Wounds with Complete Fracture of the Bone Shaft.*

1. Femur. . . . . . . . . . . . . . . . . . . . . . . . . . 24
   with no complete failure and 2 partial ones.
2. Bones of the Leg. . . . . . . . . . . . . . . . . . . . 64
   with 2 complete failures and 3 partial ones.
3. Humerus. . . . . . . . . . . . . . . . . . . . . . . . 47
   with 1 complete failure and 3 partial ones.
4. Forearm . . . . . . . . . . . . . . . . . . . . . . . . 26
   with no complete failure and 2 partial ones.
5. Clavicle . . . . . . . . . . . . . . . . . . . . . . . . 3
   with no failure.
6. Ulna alone. . . . . . . . . . . . . . . . . . . . . . . 24
   with no complete failure and 3 partial ones.
7. Radius alone. . . . . . . . . . . . . . . . . . . . . . 29
   with no complete failure and 2 partial ones.
8. Fibula alone . . . . . . . . . . . . . . . . . . . . . . 46
   with no complete failure and 5 partial ones.
9. Tibia alone. . . . . . . . . . . . . . . . . . . . . . 2
   with 1 complete failure.

*Wounds of the Hand and Foot.*

1. Wounds of the hand — injury to tendons, bones or joints. 67
   with 2 complete failures, and 4 partial ones.
2. Wounds of the foot with lesions of tendons, bones or joints 55
   with no complete failure, and 5 partial ones.

*Wounds of the Skull.*

1. Wounds of the skull without injury to the dura mater (Injuries of the scalp included in wounds of the soft parts) . . . . . . . . . . . . . . . . . . . . . . . . 26
   with no complete failure and 1 partial one.
2. Wounds of the skull with injury to the brain. . . . . . . 15
   with 1 complete failure as far as union is concerned but with 3 deaths[1] and 2 partial failures.

*Wounds of the Chest.*

Penetrating wounds . . . . . . . . . . . . . . . . . . . . 7
with no failure so far as union is concerned, but with one death.
(Wounds of the chest walls are included in the list of wounds of the soft parts).

1. One of these was due to progressive meningoencephalitis. We did not suture skull wounds when the foreign body had not been removed from the brain.

It is, of course, impossible for me to give a complete history of each case. In order to avoid confusion, however, it may be helpful to give a few words of explanation. First of all, these statistics include two distinct periods. During the first, extending from July 1915 to July 1917, our service was not specialized and received all kinds of wounds, the patients not being fit for evacuation. During this period also — at least during the first six months — we were only beginning to practice primary suture, and during this period, therefore, we had the greatest number of wounds in which we did not attempt suture. Out of 853 wounds that were not sutured (mentioned above) 768 belonged to this first period and 85 only to the second period. During the second period, July 1917 to the end of February 1918, the service was changed and received only wounds of the soft parts. It is true that, by mistake, a certain number of bone wounds, a few wounds of joints, and a few fractures were treated. Furthermore, this service being larger than the preceding one, and the conditions in the army being relatively calm, it was possible for us to keep the wounded all the time necessary. The feeling of confidence in the technique used by us increased daily, which led us to extend considerably the practice of primary suturing, and the percentage is, therefore, much higher for the second period than for the first. During the first period we primarily sutured 1046 wounds out of 2336, a little more than 44 per cent., whereas, during the second period, we sutured 1616 out of 2070 wounds, nearly 79 per cent.

During the first period the wounds not sutured primarily subdivide as follows :

| | |
|---|---|
| Not operated | 216 |
| a little more than 9 per cent. | |
| Delayed primary suture | 115 |
| Secondary suture | 191 |
| together a little more than 13 per cent. | |
| Not sutured | 768 |
| about 32 per cent. | |

During the second period the wounds not primarily sutured subdivide as follows :

| | |
|---|---|
| Not operated | 118 |
| or 5 to 6 per cent. | |
| Delayed primary suture | 116 |
| Secondary suture | 133 |
| together a little more than 11 per cent. | |
| Not sutured | 85 |
| or 4 to 5 per cent. | |

A glance at the following tables shows this difference :

| | 1st Period | | 2nd Period | |
|---|---|---|---|---|
| | — | | — | |
| Total number of wounds received in hospital . . | 2336 | | 2070 | |
| Not treated surgically. . . | 216 | 9 to 10 o/o | 118 | 5 to 6 o/o |
| Primary suture . . . . . | 1046 | 44 to 45 o/o | 1618 | 79 o/o |
| Delayed primary . . . . . | 115 | together 13 to 14 o/o | 116 | together 11 to 12 o/o |
| Secondary suture. . . . . | 191 | | 133 | |
| Not sutured. . . . . . . | 768 | 32 o/o | 85 | 4 to 5 o/o |

It is extremely difficult, if not impossible, to draw conclusions from these statistics with regard to the percentage of wounds that ought to be amenable to primary suture, and of those that ought to be united by delayed primary suture or secondary suture, or, on the other hand, of those that should be treated as open wounds until they heal spontaneously. Each one of the two periods, during which our cases were observed, was characterized by wounds of a distinctive type and by a separate stage in the evolution of the surgical technique. During the first period, which includes the last six months of 1915, we sutured from 15 to 30 per cent. of all types of wounds; during 1916 and the first six months of 1917, while still treating wounds of all types, we sutured from 55 to 60 per cent; and during the period from July 1917 to February 1918, while treating almost exclusively wounds of the soft parts, primary sutures were effected in almost 79 per cent. of the cases, but there were three other important factors which contributed to this result :

1. The time elapsing between the injury and the operation.

2. The feasibility of keeping the patient long enough in the hospital to enable us to discharge him a few days after removing the stitches.

3. The operative capacity relative to the number of wounded awaiting operation.

It is also necessary to state that in the first period of our activity we operated on the wounded almost exclusively by ourselves, whereas, during the second period, the number included the wounded operated on by ourselves and by our assistants working under our direction.

Lastly, we want to note the following points :

1. We do not include in these statistics wounds of the abdomen, where the abdominal wound was primarily sutured, for we consider them foreign to our subject.

2. We do not include joint wounds, for the greater part of such wounds were diagnosed during operation and the majority were wounds of the capsule or of the synovial membrane. It would not be fair to compare these with war wounds in general, for we have only had light cases to treat. Cases with bone lesions and those in which diagnosis was made before operation were treated in the service of Doctor Leriche.

3. The fractures included in our statistical report belonged chiefly to the first period. They were far from representative of the total number of fractures we have had to treat, for they were less serious than the rest. Lastly, the statistical report includes only wounds sutured because the cases were considered suturable, and we warn against the mistake of drawing conclusions from these statistics to be applied to wounds in general.

### Part Second.

We think it useful to give an account of the stages which gradually brought us to the primary closing of wounds and thus to relate our surgical Odyssey during the war. We must begin by stating that the circumstances were favorable. After two months of traveling — corresponding to the retreat from Belgium, the battle of the Marne, and the beginning of the battleof the Aisne — and at a period during which we had no surgical work to do, we settled in a small town a few miles from the line. There we were able to treat a certain number of gravely wounded men and to follow them sufficiently to enable us to note the outcome of our treatment and what war surgery ought to be.

### I. — *Period of Surgical Delay.*

At the start we were under the influence of the optimistic reports regarding the inocuous character of war missiles as far as surgery was concerned, and we were satisfied when we had disinfected the entrance of the wound with tincture of iodine, removed the foreign bodies which were visible, and applied a good aseptic dressing. The only cases in which we operated at once were those with vascular injuries accompanied by hemorrhage, great injuries necessitating amputation of the limbs, penetrating wounds of the skull and abdomen, and wounds of long standing with a fully developed infection. All other cases, after dressing, were placed under observation. In the majority of cases, after 24 or 48 hours, fever started, pain became more and more acute, and an abundant flow of purulent and foul fluid set in accompanied by a very violent inflammation. Surgery was then resorted to. Notwithstanding the incisions, pus appeared in intermuscular spaces, necessitating new and frequent incisions. The general condition of the patient declined rapidly. During this period gas gangrene and secondary hemorrhage were frequent, and, even in the most favorable cases, suppuration persisted. The wounded man was sick for a long time and was not in condition to be transported, and very often became a cripple. This nefarious period lasted from October to the middle of November 1914.

### 2. — *Period of Incision.*

In view of these facts we no longer dressed the wound, but operated immediately on all patients without waiting for the symptoms or appearance of infection, firmly believing that their wounds were inoculated although there were not as yet any clinical signs of infection [1].

In what did this operation consist? Incision of the wounds, removal of foreign bodies (projectiles, fragments of clothing, etc.) and packing of the wound with sterile gauze. This period corresponded to the second half of November and the first half of December, 1914. It marked a great improvement. At this time, however, we observed in our wounds a tendency towards suppuration with elimination of muscle, fascia, and bone splinters, accompanied by fever lasting a fortnight; this condition necessitated changing the dressings two or three times a week. For dressings we used exclusively sterile gauze and never irrigated the wounds.

### 3. — *Period of Wound Trimming.*

We thought it might be possible to do away with this elimination attended by suppuration, by the removal of the foreign bodies and all dead tissues, or tissues destined to die. The operation consisted in following the projectile along its track through the tissues and in removing the skin, the connective tissues, the fascia, the muscle, the bones, and all that experience had taught us was doomed to elimination, or that provoked infection or kept up suppuration. We ended the operation by packing with sterile gauze. This period corresponded to the second half of December 1914 and January 1915. A slight fever, between 37.5 and 38° lasted four or five days and very soon the wound became pink and healed normally.

1. During this period we received only severe injuries, rendering evacuation of the patient by rail impossible.

4. — *Period of Fixation with Iodine.*

We then began to realize that during the operation we reinoculated the wound surface. In trying to destroy by fixation the bacteria on the surface, it occurred to us to use tincture of iodine; the efficacy of which for fixation of germs in the skin had for a long time been demonstrated. In order to have fixation it is necessary that the wound should be dry, just like the skin itself. For some time, therefore, after a complete hemostasis we used hot air to dry the wound; then we abandoned this practice and merely used dry gauze firmly pressed on the wound surface, applying tincture of iodine instantly after removing the gauze. This period corresponded to the months of February, March, April, and May, 1915. We may also say that it corresponded to the suppression of pus in the wound which granulated and healed without suppuration and without loss of tissue.

5. — *Period of Carrel Method, First Technique.*

Towards the end of the month of May, 1915, we became acquainted with the method of Doctor Carrel's first technique. During the month of June, 1915, we tried his procedure, but, probably through a mistake on our part, this technique brought back the evil days of 1914.

6. — *Period of Primary Suture.*

In July, 1915, therefore, we definitely gave up the use of antiseptics and came back to operative surgery. Encouraged by the preceding period of four months, we began to use primary suture in war wounds, doing away with slow healing by granulation and spontaneous formation of the scar. The results were such that gradually and rapidly we increased the number of cases in which we practiced primary suture. In the cases in which we did not try to suture immediately, we placed stitches which were tied a few days later on, thus realizing delayed primary suture. When we were able to keep the patient long under our direction, we practiced secondary suture in the cases in which we had been unable or unwilling to close by primary or delayed primary suture. At this time we had no laboratory at our disposal and were guided by clinical signs alone, both for primary sutures and for secondary sutures. We cannot here enter into the detail of the clinical signs allowing us to decide this question. This point is, at present, only of historical interest. Often when the laboratary enumeration of organisms (by smear) is in contradiction to the clinical information, we ask the laboratory to make a culture to decide, and generally the clinical information is proved correct. We were led to insist on these successive stages of the evolution of our technique because we consider them instructive. The different steps in our technique correspond to the different stages of improvement we were obliged to pass through, and they follow each other in the course of the operation in the same chronological order as they did in the evolution of our surgical method. Each one of them must be carried out before the following one can be taken up.

In the practice of war surgery, according to circumstances, we can stop after the first operation, or at any stage, to take up later the succcessive steps when opportunity presents. We can thus enumerate at once the different steps of our technique. They are :

1. Incision.

2. Following the track of the projectile and making an inventory of the lesions produced by the same.

3. Excision of the dead tissues or those destined to necrosis, and of course, the removal of foreign bodies.

4. Hemostasis, completed by fixation of the wound by tincture of iodine.

5. Repair.

In general it is very exceptional that one is not able to take these different steps through No. 4. If, however, because of conditions at the moment of the operation, excision of the tissues is not completely performed at this time, one must, with curved scissors, complete the excision at a subsequent dressing to avoid spontaneous elimination.

When all the steps of the operation excepting the last (suturing) have been taken, clinical examination helped by the laboratory indicates with precision the moment when one can close the wound, either by delayed primary or secondary suture. We insist upon this point — that war surgery has a two-fold duty :

First and foremost, to prevent infection, and,

Second, to repair carefully and as quickly as possible the damage caused by the wound and by the operation itself.

### Part Third

### TECHNIQUE

### I. — *Favorable Conditions.*

Before taking up the technique in detail, it seems useful to indicate briefly the conditions which make the operation possible.

1. An efficient surgical organization is indispensable. It is a mistake, too often made, to believe that a war wound, contamined by the missile and the fragment of clothing it carries along with it, is fatally doomed to suppuration. Correctly and aseptically operated at the right moment, all war wounds, with very few exceptions, will unite as though they were aseptic. One must, therefore, take pains to operate with as perfect an aseptic technique as in ordinary practice.

2. Sterile rubber gloves are indispensable. It is hardly necessary to state that a surgeon in an ordinary aseptic operation takes a certain pride in not soiling his gloves by contact with the wound; there is all the more reason to avoid contact with the young colonies of organisms that are beginning to develop on the surface of the war wound. One must therefore operate with the tip of the instruments, forceps, scissors and knife, and must only use the index finger for exploring the wound in those very rare cases in which the track has been lost.

3. The assistant must take similar precautions and when he holds retractors or when he mops the blood from the wound he must avoid as far as possible the contamination of his gloves. A point on which we insist frequently is that the blood be wiped away by pressure without rubbing. Friction of the wound has two great drawbacks : (1) it contaminates the sterile parts of the wound; (2) it runs the risk of causing the operator to lose sight of the little track caused by the missile, which he may afterward have a great deal of difficulty in finding.

4. The ideal would be to set aside every instrument that has served once, and to take up a fresh one. Practically, however, if those instruments which have come in contact with the parts contaminated are wiped carefully with a piece of sterile gauze at once (smooth instruments, like knives, scissors, and retractors), one can, without great risk, use the same instruments during the operation until the moment comes for suturing.

The instruments most frequently necessary are small in number and in general for each operation a box of instruments sterilized in formalin vapors at 50° Centigrade is used. It contains: 8 Kocher forceps, 5 Terrier or Péan forceps, 4 towel holders, with sharp points, 1 grooved director, 1 knife, 2 thumb forceps, a medium sized curette, a raspatorium, 1 long forceps and 1 Rongeur forceps, 2 pairs of scissors, straight and curved. Generally the latter are of small dimensions.

When, in the course of the operation, we need supplementary instruments, we take them from a special box prepared for the purpose. Each operation requires a separate set of instruments. Sterilization by means of formalin vapors has always given full satisfaction. Three boxes of instruments are necessary for every operating table working without interruption, provided the time of one orderly is devoted exclusively to preparing them. The rotation of the boxes is as follows. One box is in use in the hands of the surgeon, another is in the hands of the orderly who cleans the instruments and prepares them, and the third is in the thermostat.

We can not too strongly emphasize the fact that the war surgeon who wishes to succeed in primary suture, must understand that it is a difficult, minute, but by no means brilliant task which he has to take up; but he may be assured that his results will be in direct proportion to the care he bestows in following minutely the track of the projectile; in investigating the injuries produced; in doing away with the culture media; in removing minutely the foreign bodies and all the bone fragments; and in sparingly excising the skin, the connective tissue, the fascia and the muscles forming what has been called the chamber of attrition. He will end his operation after careful hemostasis, by thoroughly drying the wound and fixing it with tincture of iodine, and by repairing the injuries due to the wound and to surgical operation. We can affirm that in this labor, more than in any other, success rewards effort.

5. Usually the patient requires general anesthesia, for one never knows what the wound may lead to. Local anesthesia decreases the resistance of tissues already contaminated by bacteria. Spinal anesthesia can be used in a number of cases.

6. The most delicate task consists in following step by step the track of the projectile. It is indispensable that the surgeon should see distinctly, and that the light should be so arranged that the progressively extending field of operation may be seen step by step in such a way that everything to be removed may be clearly visible. A frontal mirror with electric light may render invaluable service.

7. It is hardly necessary to say that the surgeon must always have present in his mind the topographical anatomy of the region he is operating in. The projectile does not follow ordinary surgical paths, and so it is a point of capital importance to know exactly where one is working from an anatomical viewpoint. The track of the vessels and nerves must be well in mind beforehand, since a wound starting far from them may take us gradually into their vicinity, very often by a wholly different path from that followed in ordinary surgical operations. Projectiles take no heed of classical anatomical paths in reaching an organ, and, furthermore, they often strike a bone which sends them in another direction. All these conditions present difficulties, from an anatomical viewpoint, and yet the surgeon must know how to overcome them without causing more than a minimum of damage to tissues.

This is not all. Muscles underlying each other are not necessarily at the same point of contraction at the time of the injury. At the moment of the operation, under the influence of a general anesthesia, the perforations in any one of them may not correspond with those in the others. In such cases we have therefore a broken line to follow, which creates a real difficulty for the operator. Often a matter of capital importance for the war surgeon is a knowledge of the innervation and blood supply of the muscles. We shall also have to revert to this point later on.

8. It is of utmost importance that the surgeon should have the firm determination to attain his aim.

### II. — *Investigation before the Operation.*

1. *Radioscopy.* — For a long time we operated on our patients without the help of the X-Ray outfit. We were thus thrown back on our own resources, in following the missile step by step, and we can affirm that we succeeded with comparative ease in thus removing projectiles the situation of which was unknown to us. This necessity of not losing sight of the track leads the surgeon to follow it very closely. It trains his judgment and gives him sufficient dexterity to perform easily a surgical operation which at first is not without difficulty. Thus we believe that in the immense majority of cases radioscopic examination is sufficient. It indicates about where the projectile has remained, and consequently about the direction it has traveled. It indicates the size and, if there are several, the number. All of this is not without interest. One entrance may lead to two projectiles in the tissues, especially when a bone has been hit. In this case radioscopic examination will warn the surgeon that he must follow a Y-shaped track, the bifurcation taking place at the point where the missile has broken up. In other cases it will reveal the existence of a wound or fracture which had escaped clinical examination or merely been surmised.

Lastly, in the case of multiple wounds by fragments of hand grenade, we have often been forced to do away completely with a radioscopic examination, operating on an aluminum table, aided only by directions given from time to time by the radiographer with his frontal screen. We do not, however, allow ourselves to be guided entirely by him, for here one must follow the track of the fragment, and not, as one is too often tempted to do, go directly for the foreign body by the shortest path. This method of procedure has a two-fold advantage : first, it saves time, the time necessary for the radioscopic examination; and second, it enables the surgeon to be constantly informed of the number and size of the projectiles that still remain to be extracted, both of which considerations are important, if one remembers that men with multiple wounds are often in a condition of shock preceding their infection, and that the operation must never exceed in severity the patient's powers of resistance.

### III. — *Clinical Examination of the Wounded.*

a) *General Examination.* — We believe it necessary to insist on the importance of complete clinical examination of the wounded before deciding to operate. Slight wounds and those which we know to be rarely infected can at once be eliminated from the list for operation. Such wounds are those with minute fragments in the face and hands, which cause no injury to the bone and tendons or to the joints. Wounds of this kind contain no fragments of cloth, which are always loaded with bacteria and which are far more dangerous than the projectile itself. In the same category are included seton wounds by rifle bullets when the wounds of penetration and exit are both very small, without bone, joint, vessel, or nerve injury, and when the track is neither tympanic nor painful. One can always eliminate — but for contrary reasons — moribunds and those whose general condition contra-indicates any kind of operation. It is a delicate question and it is diffficult to say when and where the surgeon must run the risk and operate just the same, but he must know at least that the first contra-indication to any kind of primary suture is a bad general condition. These cases do not form a part of our subject matter and we only mention them in order to emphasize the following principle : *Patients whose general condition is not satisfactory must never be primarily sutured.*

The pulse must be examined. It will be far better than the temperature to enable us to foresee at once whether one should perform primary suture or not. We have never sutured primarily any wound on a patient whose pulse was above 120 when he entered the hospital, but we have often successfully sutured wounds on patients who had a rectal temperature of 38.5° C.

A great deal of importance has been attached to the time elapsing between the injury and the operation; some have gone so far as to prohibit primary suture upon patients whose injury dated back more than 8 hours. Of the cases mentioned above, a large proportion were sutured more than 12 hours after the injury; in many instances 24 hours after the injury; and we are sure that in a few cases the delay was even more extended. It remains, however, an established fact that time is a factor of great importance and that the percentage of cases suitable for suture decreases as time elapses after injury.

b) *Local Examination.* — Local examination will also give us information.

1. Not very long ago we drew the attention of surgeons to the importance of recognizing gas infection before the operation. This can be accomplished by nail percussion (filliping) and we advise strongly that this examination be made systematically in the region surrounding the wound. Very often one is surprised to hear a tympanic sound, which symptom this method of investigation alone discloses. The tympanic region may extend far from the entrance of the wound, from the projectile, and from its track. We have noticed also that this mode of investigation frequently produces intense pain at the point of greatest tympanitis. Research and the injuries invariably found in the course of an operation have proved to us that these tympanitic wounds attended by pain always correspond to gas infection by anaerobic germ. What action must we take with regard to primary suture? We advise, at least until full information is obtained, not to suture these wounds primarily. They are, however, in the majority of cases, suitable for delayed primary suture. We must say that amongst the 2537 cases primarily sutured, as reported above, there were more than 200 in which we did not find this symptom before operation and in which, during the course of the operation, we found and excised gelatinous infiltration of a pink color in the connective tissue, both subcutaneous and intermuscular, and yet these wounds united without suppuration, the way an aseptic wound would have done.

2. We must also mention the importance of a clinical examination in the search for motor or sensory paralysis. This investigation allows the worker to diagnose before operation all nerve lesions which should be repaired whenever possible.

3. Vascular lesions, which are often overlooked, must likewise be thought of. They may influence the technique followed in the pursuit of the wound track and likewise the decision taken at the moment of closing the wound. We may say at once that it is an absolute rule never to suture a wound below another wound which has caused an injury necessitating the ligature of the main artery which irrigates the region in question. Let it be well understood that the great risk in primary suture — gas gangrene — finds its principal factor in the existence of necrotic or badly irrigated muscles, and only a secondary factor in their contamination by anaerobic germs.

A war wound cleared of this necrotic tissue, the cells of which are copiously irrigated by oxygenated blood, can be sutured. The surgeon is warned in sufficient time, by symptoms of which we shall speak later on, to remove the stitches and pack the wound.

So much for the wound underlying the arterial lesions. What position must be taken with regard to the wound which has caused the lesion? The factor of the general condition due to hemorrhage being eliminated, two types of injury must be considered :

*a*) In the first the limb may be infiltrated with blood, tense, almost double in volume, an abundant hemorrhage having taken place in the depths and produced an internal stoppage of the circulation. In that case no suture must be attempted. It is often necessary to split widely, even to split the muscle fascia distended by this hematoma. The muscle must be freed, and every source of compression removed, since all interference with the circulation creates the danger of contamination by anaerobic germs, thus rendering it a prey to gas gangrene because of the enormous decrease in its vitality and because of the lack of oxygen.

*b*) In the other type of injury the arterial wound has caused but little hemorrhage. It is really a dry wound, The limb is not distended. There is no diffuse hematoma. Under these conditions the wound may be sutured.

4. Clinical examination will also give us information concerning muscular destruction and bone injury.

Certain wounds of the soft parts present such an appearance that from the start every surgeon knows that he can not — and that, even if he could, he should not — perform primary suture. The same holds true in the case of fractures.

5. There are also frequent cases where the patient has multiple wounds. It is perhaps chiefly in these cases that the clinician must take precedence over the surgeon. Sometimes he may find the patient's power of resistance greatly reduced and he will hesitate to add the further shock of an operation. In other cases he will be obliged to decide which of the wounds demand immediate operation. In such cases he will limit himself to a careful laying open of the wound; sometimes he will even omit to operate less important injuries which he would have operated completely and sutured if they had been single. All this the clinician can learn from examination of the wounded before taking up his instrument, but even in the course of the operation the surgeon must rectify the initial decision, deciding for or against suture according to the aspect of the lesions which he meets in the course of the operation.

### VI. — *The Operation Itself.*

1. *The Incision.* — The first step of the operation consists in incising the skin, excising at the same time the cutaneous border of the wound and the track in the subcutaneous connective tissue down to the fascia.

If in peace time surgical operations may usually be carried out as planned, the surgeon knowing from the start what he will have to do, such is by no means the case in war surgery. In civil practice one knows from the start the length and direction necessary for the incision and only very exceptionally is one obliged to prolong or to deflect it in the course of the operation. In war surgery, on the contrary, one does not know beforehand what lesions or infiltrations will be met with in the course of the operation. Guided by data from the clinical examination and by radioscopic examination with regard to the direction of the wound track, its depth, and the underlying organs, the operator decides on a general direction, commonly parallel to the axis of the limb, and on the length of the incision which at the start will be relatively limited.

Having immobilized the skin in a position which brings the skin wound exactly over the opening of the track in the underlying sub-cutaneous tissues, we excise a small oblong skin flap with the sub-cutaneous tissue, by two slightly curved incisions passing about one centimeter from the lips of the wound, and joining each other approximately three centimeters from the ends of the wound. Suture performed in this fashion enables us to get a linear scar.

We insist that it is not necessary to excise very much skin. Generally one centimeter beyond the wound is sufficient. It is, however, necessary in certain cases to be less sparing in this excision. Let us repeat it, economy of tissue must be the rule of the war surgeon. In removing skin, as well as in excising muscle, the operator should not treat a war wound as he would a malignant tumor, that must be taken out *en bloc* and as extensively as possible. We cannot, however, insist too strongly on the fact that dead tissue must be removed as well as tissue destined to die. But only such tissues need be removed. We are convinced that in the matter of removal many surgeons, who at first sinned by being too conservative, are at present sinning in the opposite direction.

We remove the skin and underlying adipose layer in the initial portion of the track taking care that no instrument becomes contaminated. We now have a little gap five or six centimeters in length, except in cases where a large skin wound has already obliged us to make a greater excision. Exceptionally, however, we give it greater dimensions at the start. We prefer increasing it as the need arises, inwards or outwards, transforming it into a flap as in the course of the operation the necessity or utility arises. In this way the final incision will never be greater than necessary.

2. *Following the Path of the Missile.* With a few clips of the curved scissors we remove, if necessary, a few fibers of connective tissue, expose the fascia, and easily recognize the perforation through which the projectile has passed.

Two clips of the curved scissors, often very economical, increase this opening and remove its debris. Two Kocher forceps grasp the lips of the cone-shaped opening, allowing us to observe for a certain depth into the muscle the path followed by the missile. The split fascia and underlying muscle in the direction of the track allows us to see better and to bring to the surface this part of the tunnel. If bleeding occurs it is immediately sponged. The small arteries are ligated with very fine catgut, the strands being cut very short. This can often be done by a single knot. If the ligature has included muscle tissue, we may often remove with the scissors the small stump thus formed.

We note the aspect of the muscle surrounding the track which has been laid bare. One may thus obtain a clear view of the track through the first muscular layer. With the aid of sharp curved scissors, we excise this muscle, removing only dead muscle fibers which do not bleed, which have lost their normal color, and which do not react by fibrillary contraction when pinched with the forceps. It is well understood that we will have to remove in the course of the operation all foreign bodies and fragments of clothing which are disclosed. Hemostasis is then carried out. Retractors spread the wound and very often one sees the track of the projectile continuing in the underlying muscle. Retractors expose to the eye and to the instrument of the surgeon greater and greater depths of the wound track. Very often we have followed a projectile the size of a pea through the thigh or through the buttock down to the bone through a cutaneous opening of not more than eight to ten centimeters and we affirm that with careful hemostasis one can complete the operation in the immense majority of cases. When we deal with a seton wound, situated, for instance, in the thigh, and occupying the greatest diameter of the limb, we follow the track through one of the wounds, the one which appears to be the least traumatized; then, about midway, we leave a small gauze packing and start at the other extremity until we reach the gauze. In a case where a projectile has gone almost through the thigh but has lodged a few centimeters from the skin on the opposite side, we follow the track from the entrance until we reach the axis of the limb and, after opening on the opposite side, we go directly to the projectile and continue until we reach the path we first excised. This following of the track is often easy, but not always so. It is possible to lose one's way, and in this case one must avoid above all things the use of the grooved director for exploration. It will merely create artefacts.. By careful inspection of the point where the track has been lost sight of, one has the best chance of picking it up again. It will, appear in the shape of a little blood clot or an adherent fragment of clothing, or a single ecchymotic spot in the muscle. By lifting a few muscle fibers and by drawing them a little sideways, one may find the way again. If we lose the track at the deep surface of the muscle, let us recall the physiology of the underlying muscle and see if shifting of the two muscles has not caused a broken line such as we mentioned. Let us therefore retract a little above and below the first muscle. If we ask the assistant to impart a few slight movements to the limb we will often have the agreeable surprise of seeing the lost track reappear. If all this leads to nothing, let us find the nearest edge of the muscle, detach its surface from the underlying tissues and thus we will find the injury in the underlying muscle. We must, however, be careful not to infect the sliding surfaces of the two muscles, for sometimes we will discover at this point a few fragments of cloth left by the projectile and dispersed by the sliding muscle.

Sometimes things are still more complicated. Let us suppose the case of a projectile which instead of traveling in a perpendicular direction to the axis of the limb, has traveled in a longitudinal direction We begin as follows : After following the track as far as possible through a sufficiently large but not excessive opening, we mark the point where we stop by a little gauze mop, and then, by a second incision parallel to the first one, we go back to this gauze mop and continue the pursuit of the wound track. If this second opening does not allow us to reach the projectile, it is very rare that a third incision, taking us directly down to the foreign body, does not allow us by retrogression to find the second stopping point, thus exposing the whole wound track. Several times we have utilized these ladder incisions and our objective has always been obtained. We can not too often repeat that the track must be followed, that it must be seen in its entirety, and that the operator must pass wherever the projectile has passed. This is one of the important factors of success in primary suture. An operation may succeed, perhaps, in other cases in which the track has not been followed, for the whole extent of the track is not contaminated. We have noted that the wound track was contaminated by fragments of clothing, especially in the first few centimeters of its length and where it went through the fascia and bone. Sometimes, in order to avoid anatomical injuries out of proportion to the gravity of the lesion, we wipe out part of the track with a sterile piece of gauze, pulled through by forceps, in the direction supposedly followed by the projectile. At the time of closing by suture, and also as we watch the patient after the operation, it should be remembered that this slight deviation from the rule has been made.

3. *Inspection of the Track — Inventory of the Damage Done — Excision.* We have now followed the wound track completely. We have already removed all foreign bodies and bone splinters, if there were any. We have excised the cutaneous wounds. We have removed the dead muscle and excised other tissues when necessary, and investigated all the damage done.

*a*) Nerve lesions should be repaired immediately, by suturing any nerve that happens to be cut. If the nerve wound is only partial without complete section it is sometimes well to reinforce the union by a stitch bringing together the neurilemma. Unless there is a special indication, such as the proximity of fracture, we do not enclose the nerve in muscle or other protective tissue.

*b*) Vascular injuries will be treated later on.

In the majority of cases double ligature will be necessary, but we must take care to place the ligature one centimeter above the injury and to excise the injured portion of the blood vessel. A vessel can seldom be sutured. However, in small lateral wounds we recommend suturing the vessel provided the surrounding tissue is healthy. But we know the danger from projectiles in the region, which may produce secondary hemorrhage and cause the development of aneurism, and we must therefore be very circumspect. Very often arterial lesions, even if important vessels are concerned, are disclosed only in the course of the pursuit of the wound track. They may have bled very little, the perforation being momentarily and very rapidly obliterated by blood-clot or reflex vaso-constriction or, in the cases of complete section, by retraction of the middle tunic. They became manifest in such cases when we detach the blood clot covering the opening. Often it is possible to treat these lesions easily through the path followed; but sometimes one meets with very great difficulties which, not infrequently, it is impossible to overcome. Therefore, we always have in our service a tourniquet within reach of each operating table.

Often, also, the artery lesions are recognized at the time of the linical examination. From the viewpoint of the technique that is to be carried out, two cases may occur. Either we may consider hat the direction of the track will enable us easily to expose the arterial injury and we may follow this path, or else we may consider that this path is impossible or too risky, and so begin the operation by an incision which leads us directly to the artery. After the artery is treated we suture this aseptic wound primarily. Then, as though there had been no arterial injury, we treat the track of the missile.

When the lesion affects a large venous trunk we incise in the same way, but in such a case the tourniquet is unnecessary : compressive packing of the wound is sufficient until the ligature has been completed.

*c) Bone Injuries.* — It is necessary to remove all splinters. With the Rongeur forceps we must freshen the bone wound when there is only an incomplete fracture. When there is a complete fracture it is necessary to remove the splinters as completely as possible, taking particular pains to preserve the periosteum and the underlying osteogenetic tissue, the importance of which has been so well emphasized by Doctor Leriche. It is necessary to inspect the bone marrow to remove that which is in the vicinity of the focus of the fracture, but not to remove the marrow extensively, as we have sometimes seen done.

Articular bone lesions must be treated according to their importance, either by classical excision of the joint at once, when it is possible, or by regularization of the focus of the fracture.

We do not want to insist on this point as it would involve lengthy explanations and we can only repeat what has been said by Doctor Leriche on the subject.

*d*) We think it is advisable, on the contrary, to insist on what must be done in the way of muscular and fascial excision.

In general the aponeurosis, which is resistant, is simply perforated by the projectile. Sometimes the tear extends a little beyond the perforation which is always fringed by shredded tissue. These shreds must be excised for a distance of a few millimeters, but the most important thing is to inspect them carefully, for often one finds among them minute fragments of clothing adhering to the tissues which may become most important factors of infection. Intermuscular fascia must likewise be inspected with particular care and we advise the removal of a little layer of muscular fibers, often healthy, which adhere to the fascia at this point, in order to have a clearer view and so detect those little fragments of clothing which are greatly to be feared. Sometimes it is necessary to inspect both faces of this tendinous layer, to split above and below the opening and to turn out the two margins with the Kocher forceps, thus exposing the deep surfaces to the eye of the surgeon. This is likewise necessary to enable one to follow the track later on.

*e*) Tendons which are in the way of the projectile must likewise be minutely inspected. When they are cut they must be sutured after freshening. When normal suturing is impossible they must be sutured to a neighboring tendon; in short, one must perform immediately the operation necessary for the best functional repair of tendons. If they have only been brushed by the projectile they must be cleansed of minute fragments of clothing which may adhere to them. These wounds must be treated the way old surgeons used to treat an amputation stump, and they must be reinforced with a catgut stitch if this treatment has increased the initial injury and rendered them dangerously weak.

Sometimes, in wounds of the wrist and ankle, we have had to dissect so as to cleanse and repair the extensor or flexor tendons the way we would have done for the removal of tuberculosis synovitis, and thus to obtain after primary suture a perfect functional repair.

*f*) We must not forget to excise as completely as possible any sub-cutaneous or intramuscular tissue infiltrated by dark blood or transformed into a kind of pink or brownish green gelatinous substance. Surgeons do not like to meet with this condition for it is often a prelude to grave infection and accompanies generally the tympanitic tracks already mentioned.

*g*) Lastly, the muscles (soft tissues gorged with blood) explode very often under the influence of the impact. The irregularities of the artillery projectile, and especially the enormous speed which all war projectiles acquire, produce in these tissues lesions of explosion and commotion paralyzing and devitalizing these organs which are of a relatively delicate texture. Experience has shown that grave infection occurs in war wounds owing to a twofold factor: 1. mortification of the muscle which becomes an excellent culture medium in the well regulated thermostat of the human body; 2. introduction of micro-organisms by fragments of clothing carried by the missile. The first of these factors is by far the most to be feared, and the second without the help of the first is very often not able to produce its effect.

We must therefore remove all the necrotic portion of the muscle, but only such tissues as constitute the culture medium. Certainly, in the beginning, the surgeon must hesitate in determining exactly what he must remove and what he must leave. Sometimes he removes too much, sometimes he does not remove enough, and it is only his personal experience which will tell him, as his judgment improves little by little, the exact line of separation between the two. It is a question which varies according to the nature of the case, and varies also with the nature of the wound and the depth of the track. There are some cases in which any muscular excision is unnecessary. There are others in which it is necessary only at the extremities of the track. There are others in which the removal of a few fibrils may be sufficient, but there are others in which it will be necessary to remove considerable masses of muscle; in certain muscles already the prey of gas gangrene, whole muscles at a time. There are, however, certain signs which may guide the surgeon. These signs are three in number:

1. Normal coloration of the muscle;
2. Bleeding of muscle, when tested by a minute clip of the scissors;
3. Fibrillary contraction, when the muscle is stimulated by pinching with the forceps.

Sometimes the muscular tract presents normal coloration. It is sufficient, then, to inspect it to remove little fragments of cloth that may exist, and to freshen it by a few scissor clips. In this case, muscular excision is practically unnecessary. Very often the track is of a grey hue, even black. In such a case the superficial layer of the track must be removed. In general, unless there is gas infection, it is unnecessary to remove a thick layer; half a centimeter is often sufficient, sometimes less. It is rarely necessary to remove more. In those cases we recommend careful examination of the fresh section of the muscle thus produced, to see whether there is any open space between the muscle fibers, or any cut fibers the extremity of which may have carried back with them into the body of the muscle little fragments of clothing.

Sometimes one finds the muscle paler than usual — pinkish in hue or copper-colored, and very often in this case the first excision, always performed with curved scissors and about one centimeter from the surface, produces a section which presents the same aspect.

The blood oozing on the surface similar to a pinkish dew is not to be seen. True, we may see a little blood jet from a little intra-muscular artery which we have cut, but this is not a sign enabling us to say the muscle is normal. Moreover, if the muscle is stimulated by pinching slightly with the dissecting forceps it does not react by the little fibrillary contraction which is so characteristic.

What should we do in such a case? We advise splitting the fascia of the muscle about which we are in doubt, giving it breathing space and allowing it to spread out freely; then we would wait a few minutes, taking up another part of the wound. One is then surprised to see this muscle, which was pale a few moments ago and which did not bleed upon section or react when it was pinched by the forceps, offer now simultaneously these three symptoms. The muscle was apparently suffocated and the scissors, in freeing the tissue, allowed it to recover life. It is really a live muscle and its excision would have been useless.

If, on the contrary, after this trial, it does not manifest its vitality we must excise progressively, still using the curved scissors and taking off a layer of a few millimeters at a time until the necessary characteristics appear beyond a doubt. That is precisely the reason why we insist that the quantity of muscle to be removed varies essentially according to the case and we appeal to the judgment of the surgeon, who, thanks to these symptoms, very soon acquires sufficient experience, to decide what must be removed and what may be left.

In other cases the small artery has infiltrated a muscle with blood. With a few clips of the scissors we detach the principal clots. It is often impossible to leave a few dark spots resulting from this infiltration of blood without risk of compromising the primary suture.

We have already specified, but deem it necessary to repeat, that by bleeding muscle we must not understand bleeding from a cut artery, but that little capillary oozing which appears like a pinkish dew on the whole surface of the section.

We will not insist any more on the sign of fibrillary contraction in response to stimulation by pinching. It must be constant at the proximal end of muscular fascicles which we examine; but it may fail at the distal end, being dependent on the condition of the nerve supply. Let it be well understood that the important thing is to free the wound of all devitalized tissues. We must therefore leave in the wound no fragment of dead muscle and instruct our assistant not to traumatize with retractors or Kocher forceps the delicate muscular tissue, the vitality of which is easily compromised, creating one of the important factors of grave infection following war wounds.

One has doubtless noticed how much we insist on the use of curved scissors. They must cut sharply, not with their point but with their blades, cutting at a tangent to the surface and for this work they are infinitely superior to the knife. The latter may prick with its point and thus inoculate, and the stimulation it produces in the muscular fibers it cuts often causes in the underlying ones a reflex contraction. Sometimes it even carries with it into the depth of the muscle little fragments of clothing. We apologize for stating once more that muscular excision must be economical; muscle may compensate and adapt itself to a new function, but it does not regenerate. Therefore, in the immense majority of cases — we would almost say in all cases — one must avoid cutting the muscle transversely. Such incisions interrupt not only the muscle itself, which may be restored by primary suture, but also the nerves and the blood supply to the inferior portion of the limb, thus creating the possibility of serious consequences. It is obvious that the distal end will no longer receive its proper nerve supply, and suppression of the arterial blood jeopardizes the vitality of the distal end, sometimes causing necrosis, accompanied by gas infection and, at least, causing a failure of the primary suture. For instance, we know how grave from a functional viewpoint are transfixing wounds of the calf, and yet we can affirm that if they are judiciously treated they almost always heal by primary suture. The difficulty the surgeon so frequently meets in these cases is due to a two-fold cause — the blood supply of the gemelli muscles and the presence of the thick intramuscular aponeurosis of the soleus muscle. The large muscle bodies of the first named are supplied almost exclusively, each one independently, by a single artery. If one of these arteries is cut, either by the projectile or in the course of the operation, the vitality of this muscular mass is so much lessened that the worst disasters may be feared. One must abstain from primary suture in all such cases.

As to the intramuscular flat tendon of the soleus, it is situated in a deep portion of the limb, concealed by a thick layer of tissue and the projectile often carries to this point the fragments of clothing.

One sees at once what risk would be incurred in a wound of this region, if it were sutured without the surgeon's being certain that no foreign body had passed unnoticed.

If, notwithstanding, in certain cases one feels compelled to cut a muscle crosswise, one must not do so unless it is a muscle receiving its nerve and blood supply at several points, so that one can afterwards by suture restore its anatomical continuity. In a majority of cases it is preferable, if feasible, to work round the muscle, passing in that way the obstacle which it presents, and taking up the track through this new path, without omitting the examination of the small portion of the track that could not be investigated by the direct path.

We admit, however, that there are cases in which one must expect functional sacrifice of muscle, but at least it is a very rare exception indeed, and only to be made when cutting crosswise would involve a still graver risk. Two words sum up what this excision of muscle tissue ought to be: no stinting; no prodigality.

The same general technic is to be recommended, whenever it is practicable, for the treatment of all the tissues and even of the viscera; that is, resection of the edges of the wound before suture. In this connection penetrating wounds of the cranium require special mention; here, as elsewhere, it is necessary to eliminate the portions of cerebral substance injured by the projectile. Nothing could be easier: all that is necessary is to require the patient (whose wound has just been opened and cleaned under local anesthesia) to cough. The simple effort of coughing, which increases the intra-cranial tension, causes the contused cerebral matter to issue from the wound, for its liquefaction is always extremely rapid. This action repeated two or three times, at intervals of a few seconds, produces by itself the necessary ablation, and renders the wound suturable.

4. *Drying and fixation with tincture of iodine.* The investigation we have made has allowed us to determine the unavoidable losses of tissue and to clear the situation. Let us now say a word with regard to the best means of repairing without risk to the patient.

As we have said hemostasis has been insured in the course of the operation. This hemostasis must be very thorough for two reasons: first, it must prevent the formation of hematoma, which favors suppuration; second, the wound must be completely dried so that its surface can be fixed by the action of tincture of iodine. We therefore dry each wound completely with gauze pressed on the surface and rapidly removed just as the wound is to be abundantly swabbed with tincture of iodine. Immediately another dry gauze mop wipes off the excess of tincture of iodine. If the fixation has been well done, the whole wound surface becomes dry as though varnished and assumes a coppery hue which is absolutely characteristic. This procedure, if correctly carried out, is of great importance. Certainly, we do not pretend that a wound which has not been treated in this way will necessarily suppurate. Numerous facts would contradict our statement at once, and we do not resort to this mode of action ourselves in simple cases, where the pursuit of the wound track has shown us healthy tissues which require no excision, or in superficial wounds, which are totally excised. We have already explained how we were led to adopt this treatment. We believe, indeed, that the small number of failures, shown in our statistical report, is due, partly at least, to the careful carrying out of this step of the operation.

We took up tincture of iodine (5 o/o) as fixative agent because it was used before the operation for the fixation of the superficial layer of the skin and also because it is easily applied and always at hand; but we are ready to abandon it for any other fixative agent which offers more advantages, and which is free from the drawback we are about to mention.

Tincture of iodine fixes not only the superficial micro-organisms but also the underlying superficial cells of the wound. As a result a slight secretion of turbid serum takes place, which oozes out between the stitches of the wound or along the capillary drain when one is used, but which produces no harm, not even requiring a renewal of the dressing, as it drains into the deepest layer of the same. When capillary drainage has been used, it is necessary at inspection to remove this drainage only on the third or fourth day. Furthermore, the slight serous elimination delays the cicatrization for four or five days. For this reason we advise the removal of the stitches as late as the 12th or even the 13th day.

This slight drawback has an advantage : it permits the postponement of the primary suture, when thought best, till the eighth or ninth day. We have had some cases of delayed primary suture performed on the tenth and twelfth days.

5. *Suturing.* — As the terms " primary suture ", " delayed primary suture ", and " secondary suture " are coming more and more into use, it is necessary to understand clearly the meaning attached to each of them, to avoid designating the same process by two different names. For our part, we understand by immediate primary suture the normal termination of the operation we have just described. The suture takes place immediately after the excision and treatment of the wound.

Delayed primary suture, without further excision or freshening of any kind, consists in the repair of anatomical layers, when the gap of the fascia is not too great. The operation is taken up after a varying interval of days, and is carried to completion.

On the contrary, secondary suture consists in closing the wound after excision either of all the scar tissue in one mass or of only an epithelial border or a narrow strip of skin in the region of the wound. As little as possible of the granulation tissue should be excised.

We therefore think it advisable to distinguish two kinds of secondary suture. In one, the scar tissue is removed as a whole; all the layers of the fresh wound thus produced are reconstructed. This might be called secondary suture with anatomical reconstruction. In the other, which might be called secondary suture of the skin, the operator confines himself to excision of the epithelial border allowing the granulation tissue, entirely or in part, to remain.

We always suture the wound primarily when we deem it advisable; otherwise, we follow our patients clinically and bacteriologically with a view to delayed primary suture. When we cannot perform the latter, we endeavor to perform secondary suture as soon as possible. In these cases we prefer an anatomical repair of the wound, except when it is impossible for one reason or another, and in this last case we perform a secondary suture of the skin alone.

We do not propose to discuss the technique of secondary suture. The only reason for mentioning it was to emphasize the following fact: When we cannot suture primarily, the treatment of the wound consists in a simple, dry aseptic dressing, without the use of any antiseptic, the dressing being renewed every five or six days. At each dressing it is necessary to make sure that there are no necrotic tissues to be removed; should there be any they are detached by a snip of the curved scissors. Then we wash the skin surrounding the wound with oleate of soda, we dry with a gauze mop imbibed with ether, and paint the surrounding skin with a little tincture of iodine.

We can affirm that wounds treated in this manner suppurate very slightly if at all. Sometimes during a week in the interval between two dressings the wound is somewhat grayish but very soon becomes pink. In a word, when primary suture and delayed primary suture are both impossible we trust to the vitality of the patient for the disinfection of the wound without striving to destroy the micro-organisms, leaving this to the phagocytosis but taking care not to interfere with the process of auto-immunization of the patient.

Laboratory examination can be used to ascertain the cellular and microbic condition of the wound, rather by means of cultures than by smears. It is the general condition of the patient, the aspect and evolution of the wound, and the absence of streptococcus that will enable us to decide when it is possible to suture [1].

We are convinced that treated in this way our patients are ready for secondary suture as early as if they had been treated by the Carrel method.

It is unnecessary to describe in detail the technique of the primary suture of the wound. Suffice it to say that the different anatomical layers are repaired whenever possible, and that security can be increased by using the capillary drain, composed of three or four silkworm guts which are removed on the third or fourth day; but there are many cases in which we do not place any at all.

It is useful to note that, for the repair of the different layers with buried sutures, one must use nothing but fine catgut, on the principle that every foreign body and animal tissue favors suppuration of the wound even if it contains very few organisms. For the same reasons, we cut the strands of the ligature very short and avoid strangulation of the tissues.

We end by applying dry sterile dressing and immobilizing the operated region as much as possible.

### V. — *Post-operative care of the patient.*

This is very simple. Unless there is a special indication to the contrary, the dressing is changed at the end of twelve or thirteen days, and, if there is no capillary drain, the stitches are removed. A drain may be removed at the end of three or four days. In such cases a second dressing is necessary when the stitches are removed. In the great majority of cases the wound unites by first intention and after the fifteenth or eighteenth day no more dressing is required.

We must also mention that after the suture the mobilization of the operated region must be avoided, for, as we have already said,

1. A longer experience now permits us to say that the streptococcus does not always merit its reputation for danger. About fifty wounds operated by Doctor Lemaître, in which the bacteriological examination showed the presence of the streptococcus, evolved in a perfectly normal manner.

we close wounds which are not sterile from a bacteriological point of view. Their evolution is that of a sterile wound because we have removed by operation the great factor of infection, and the defense of the organism does the rest. Nature has foresight and realizes immobilization of an infected region. We must imitate it in immobilizing the region we have operated upon. The results of primary suture would not be so good were one obliged to evacuate the patient and make him travel prematurely. However, this period is relatively short and four or five days after the removal of the stitches the patient may travel. Many of our wounded leave the hospital for convalescence around the twentieth day, sometimes even a little earlier.

Let us now see what points deserve attention in the days that follow the operation. Three points are important : temperature, pulse, pain.

Very often there is a certain rise in temperature — 38, 38.5 rectally during the first two days, sometimes during the first three days; then rapidly the temperature falls to normal. We must say that certain of our cases reach a temperature of 39 degrees. This does not trouble us especially and nothing unfavorable takes place. Only a *prolonged* rise of temperature calls for an examination of the wound. If in examining the wound there is found a little redness over one of the stitches, gentle pressure over the surrounding zone is made. If this pressure is painful, it is better to remove the stitch. If, on the contrary, pressure is painless, we can leave the stitch in.

The pulse of the patient gives more reliable information. In general the pulse ought to be normal, between 72 and 80. If it is more frequent, even without rise in temperature, one must watch the wound very carefully, but one need not be alarmed. Generally the pulse becomes rapidly normal. If not, other symptoms (pain, temperature, general condition) warn the surgeon that something abnormal is taking place in the region of the wound.

We attach greater importance to the symptom of pain. In principle, twelve hours after the operation the patient ought no longer to suffer. We do not refer, of course, to pain produced by motion or pressure of the wounded part. It is spontaneous pain which calls for attention, particularly when the patient finds that the dressing is too tight. When a patient complains, making due allowance for his nervous susceptibility, we may remove the dressing. Often one discovers the cause of the complaint to be hematoma, in which case stitches must be removed, the bleeding vessel sought for ligature, and the wound packed with gauze, — delayed primary suture being performed two or three days later. In other cases we have to do with an incipient infection and then all the stitches must be removed, the wound completely laid open and packed.

This spontaneous pain is, in our judgment, the chief symptom indicating that primary suture was unduly performed.

### VI. — *Contra-indications.*

This is the most delicate point in the method. We mean the discussion of the indications and contra-indications for the primary suturing of war wounds.

In the first place, we must say that all, or almost all, war wounds which can be correctly treated in due time should be sutured as soon as possible. In other words, a suture is indicated in all cases. We shall not therefore speak of indications, but of contra-indications. These are based on manifold considerations.

Some have no relation to the patient, or to the wound, or to the surgeon; they are not based on technical reasons but rather depend on the material circumstances which cannot be modified, as, for instance, when the influx of wounded is clearly out of proportion to the limited operative capacity. Contra-indications of this nature we shall leave out of our discussions.

On the contrary, we must examine with care other contra-indications of a purely technical kind. We shall divide them into two groups: First, absolute contra-indications; second, relative contra-indications.

A. *Absolute contra-indications.* — They are the following:

1. The patient reaches the surgeon at a relatively late period after the injury. The wound is already suppurating or presents a zone of lymphangitis, with a little streak of lymphangitis at a certain distance from this, and a swelling of the lymph gland in the neighborhood., We need not insist on this contra-indication, which is obvious.

2. The same applies evidently to patients who arrive sometimes, in our experience, only a few hours after their wound, but already with confirmed and extensive gas gangrene.

3. In no case, excepting for joint wounds (and often in this case the suture must be limited to the synovial membrane and the capsule) should one suture primarily when a patient's general condition is bad. But what do we understand by a bad general condition? Whenever a patient shows sign of shock, whatever may be the cause of this condition. In a majority of the cases of shock, except those due to hemorrhage or to intoxication, the wounded should be watched and given general treatment before a decision to operate is reached. A wounded man who is very anemic, owing to much loss of blood, whose pulse is 120 beats a minute or above, emotive causes of this acceleration being eliminated, should not be primarily sutured. This is the meaning we give to the words " bad general condition contra-indicating any primary suture ".

4. When one has to deal with a shattered limb, or even certain injuries of the soft parts, and hesitates about the advisability of a primary amputation.

5. We have already said that no wound, underlying another wound which involves arterial injury necessitating the ligature of a vessel and a limb, ought ever to be sutured primarily.

These are the absolute contra-indications, and it is clear that they can be reduced to these general ones : Either the wounded presents an infection too advanced to enable one to think of primary union, or else the circulation, general or local, is so impaired that by suturing the wound one would cause the patient to run the greatest risk by creating a favorable condition for the development of anaerobic germs.

B. *Relative contra-indications.* — Let us now see what contra-indications may exist in individual cases. It is here especially that the tact and clinical sense of the surgeon must guide him and here the clinician can, in certain cases, stop the hand of the surgeon who might be too eager to close the wound at once.

These contra-indications are numerous. Let us examine them separately.

1. We have already mentioned the time elapsed after the injury as an important consideration in the examination of the patient before operation. We do not attach great value to this factor in deciding what is the latest moment at which primary suture ought to be practiced. Certain surgeons have spoken of eight hours as being the limit. We are not of this opinion. There are cases in which one must not suture primarily even if one can operate within the first two or three hours. There are others in which one may suture much later, about the twenty-fourth hour. However, the result of the study of the flora of the wounds shows in an unmistakable manner that the longer the time which has elapsed since the injury, the greater the importance of the micro-organisms; and it is therefore natural to take into account the time that has elapsed since the injury : but we cannot and will not fix any precise moment.

2. The same applies to the temperature taken before the operation. At the start, we never sutured any patient whose temperature was above $38^0$ C. Our personal experience, however, has taught us that one can with impunity close the wound in a patient whose temperature reaches $39^0$, provided there be no other contra-indication. These patients must be followed with special care and the fever registered before the operation will really be of more importance in guiding the surgeon after the operation than it was at the moment of the suture itself.

3. Does the presence of a painful, tympanitic zone around the wound before the operation and of a gelatinous and pinkish or greenish connective tissue at the time of the operation constitute a contra-indication to primary suture? When the surgeon is certain that he has seen the whole of the track, and has excised all this gelatinous edema and has found tissues with the normal characteristics of vitality, — in a word, when the surgeon is well satisfied with his work, — the wound may be closed, but we urge the exercise of great prudence and, until he has gained experience, we advise the surgeon to resort to delayed primary suture in such cases.

4. We have already set forth our position with regard to wounds which have produced an arterial lesion, and we have stated that they may be sutured primarily : first, when the general condition of the patient is favorable; and, second, when the wound is not infiltrated by blood. When the wound is infiltrated by blood it ought not to be sutured — rather because of a desire to preserve the nutrition of the limb than because of fear of infection.

5. Suture should not be performed in cases in which one has found in the course of the operation any lesions of explosion, any mortification, or any large quantities of clothing. One should not forget, however, that it is often possible in these cases to perform a delayed primary suture if they have been correctly operated. It is simply a question of prudence.

6. When one finds in the course of the operation that the whole missile track has a grayish appearance, it is preferable to postpone the suture.

7. When the wound is peppered with a multitude of little fragments, as frequently occurs when a hand grenade explodes in the vicinity, the vitality, if not jeopardized, is at least markedly lessened. Greater circumspection is necessary in suturing such cases.

8. When the whole wound track has not been seen, either because the guiding thread has been lost or for other anatomical reasons, one must be very circumspect. It is true that primary suture may succeed, but if it fails it is unnecessary to seek any other explanation.

9. If one is in doubt about asepsis of instruments and material used, it is obviously necessary to leave the wound open in these cases and resort to delayed primary suture.

10. When the surgeon lacks confidence in himself. This is a purely psychological contra-indication, but it has in our eyes a capital importance and we venture to give the following advice.

Let the surgeon operate as minutely and as carefully as though he intended to perform a primary suture, but in his first operations let him attempt only the delayed primary suture. After a certain lapse of time, when he sees his patients with normal temperatures and all his wounds without pus, he will regret not having performed primary suture. Having thus gained confidence in himself, feeling that he has mastered infection, he will attempt primary suture. Thus he will avoid serious disappointments.

Like the absolute contra-indications these relative contra-indications are based, as we have seen, on the antecedents of the wound, on the clinical examination of the patient, on the clinical examination of the wound, the anatomical condition of the lesions during the operation, the blood supply, and, lastly, the principle of general pathology which states that the same quantity of the same infecting organism with the same degree of virulence will produce very different effects accordingly as it has been inoculated in healthy, live tissues or in tissues that are dead or of decreased vitality.

### VII. — *Rôle of the laboratory.*

So far we have not mentioned bacteriological examination, but have placed ourselves in the situation of a surgeon not having any laboratory at his disposal.

The laboratory, however, can and must help our judgment in this matter. The laboratory test is of first importance — we may say it is indispensable — for delayed primary suture and for secondary suture. The work of Tissier has shown us how it may apply to primary sutures. In the course of a trip to La Panne, in the month of March 1917, we had the good fortune to meet him, and we remember the deep impression his opinion on streptococcus in wound infection produced upon us. This gave us the key to certain failures which we could not understand. In these cases clinical examination of the lesions found in the course of the operation had led us to perform primary suture, which we were later obliged to remove. Since then we have been able to control bacteriologically the nefarious action of the streptococcus. Always in our cases of complete failure, and often in those of partial failure, it was the streptococcus we found to be the cause of the infection [1].

In our partial failures, in which we found no streptococcus, we found staphylococcus.

We therefore accept fully the first part of the dictum of Tissier: that every primary suture of a wound, based on correct anatomical and clinical principles, where no mistake has been made in the operation, ought to unite, and that if union fails, this failure is due to streptococcus.

After thirty-one months of experience, however, especially when we consider the very small number of our failures, we are yet unconvinced that every wound contaminated by streptococcus is fatally doomed to suppuration, and the suture of such wounds to failure. We cannot believe that in the very large number of our primary sutures the number of streptococcic contaminations of the wound can have been so slight, and we do not deem it possible that we could have eliminated from primary suture all of such cases. While we continue our present practice, we hope that laboratory investigation will soon clear up the subject.

### Conclusions.

This method is in contradiction to prevailing beliefs held before the war. It was not conceived then that one could operate a wound already strongly contaminated by microbes which were developing, and close it as though it were aseptic. The method has, however, no original element. It is the result of a combination of surgical acts, which are logical and which have been known for a long time.

As a result of observation and common sense, the method has been perfected step by step. We have often been criticized for not publishing our technique sooner, for it is now, with the exception of slight improvements, what it was two and a half years ago. Our reasons for refraining from publication are the following:

1. A technique is taught by example, and not by books or words. It is understood and acquired much better by seeing the surgeon act, than by reading or listening to his description. Our first publications on the subject, in the months of March, April, and May, 1916, at the Medico-Surgical Society of the ... Army were not received with encouragement, although we already had the

1. The streptococcus still remains a frequent cause of failure, but we have just seen that such is not the rule. Failure is more often the result of a faulty technic than of the presence of the streptococcus in the wound.

experience of ten months and gave the histories of two hundred and eighteen cases, with but two failures. Our report, however, was premature. The trend of thought at that time was not in this direction. Nevertheless, primary suture seemed to us reasonable, and so consistent with surgical " common sense " that we thought there must be at the front surgeons who had been influenced by the same reasons as ourselves, and who were carrying on the same practice. At present this method of treating war wounds is becoming classical, and if we publish our statistics and our technique, it is merely to make our contribution to the subject, and to express our opinion on points where surgeons do not seem to agree in practice, even if they do in principle, thus creating prejudice against the method.

Opponents of primary suture claim that it necessitates a considerable excision of tissues, more, at least, than would be necessary for disinfection by chemical means. We are not of this opinion. Excision sufficient to make a primary suture successful is exactly equal to that which is necessary when the other method is used. Those who excise more excise too much.

*Report on the Working of Dr. R. Lemaitre's Service at the Bouleuse Evacuation Center.*

| | |
|---|---|
| During the period between August 2nd, 1917, and February 3rd, 1918, this service received. . . . . . . . | 1209 cases |
| Those who rejoined their corps directly numbered. . . | 26 |
| " " " " " after 10 days' leave . . | 183 |
| " " " " " convalescent leave. . . | 637 |
| Arabs evacuated to the Army Zone for convalescent leave[1]. . . . . . . . . . . . . . . . . . . . . . . . | 27 |
| Patients sent to the Medical Service after their wounds were healed and thence discharged as cured. . . . . | 17 |
| Total. . . . | 890 |
| Evacuated to Army Zone. . . . . . . . . . . . . . | 80 |
| " to Interior Zones. . . . . . . . . . . . . | 81 |
| " to Epernay (specialist centre). . . . . . . | 53 |
| Deaths . . . . . . . . . . . . . . . . . . . . . . . . | 15 |
| Total. . . . | 229 |
| Remaining in hospital. . . . . . . . . . . . . . . . . | 90 |

Of the 90 remaining in hospital at least 60 will be healed and returned to active service during the course of this month (February).

1. We are not empowered to give direct leave to Arabs on their discharge.

This gives a total of recovered cases discharged direct from the hospital of 890 + 60 = 950
or 79 per cent of the admissions to the service.

The average length of stay in hospital was 28 days (to be exact — 28.02 days).

These rapid recoveries are due to the fact that :

80 o/o of the wounds underwent immediate primary suture.

6 o/o " " " delayed " "

9 o/o " " " secondary suture.

The total number of wounds sutured in the service amounts, therefore, to 95 per cent. The other 5 per cent represent :

1. Those cases evacuated before it was possible to perform secondary suture.

2. The few cases which died.

3. The rare cases whose wounds healed spontaneously by granulation and skin growth (bacteriological and clinical examination having precluded suture).

It should be noted that among the cases evacuated to the army zone or to an interior zone many were cured so far as the healing of their wounds was concerned but could not be returned directly to the fighting line. A certain number of these needed a course of mechano-therapy or of electro-therapy (for sutured nerves), or a too long convalescent leave; others were awaiting a decision concerning change of category, while others were to be discharged from the army.

Those evacuated to Epernay comprised, as a rule, the bearers of multiple wounds whose condition necessitated the care of a specialist (eye wounds, face wounds, etc.)

With reference to the deaths, it is only fair that they should be examined carefully, for out of the total of 15, nine cases arrived in a moribund condition and succumbed soon after; four died immediately on arrival, and five within the first 24 hours (before any surgical treatment could be practised on account of their condition). Three others had been operated before coming to us. Only three deaths occurred among the cases operated in our service, and in these instances the patients were very seriously wounded. It should be added that none of the above cases had been sutured.

We should like to emphasize the following points : we used no antiseptics; dry, aseptic dressings alone were employed.

For the primarily sutured wounds the average number of dressings was three.

For the wounds covered merely with dry dressings and afterwards sutured secondarily, the average number of dressings was seven, including the two dressings following on secondary suture.

It seems to us of interest to examine procedure and results from the three following points of view :

1. The rapidity and the large number of complete cures.

2. The economy in dressing material and labor necessary, which lowers the cost of treatment.

3. The safety of the method, which has been practised by the Chief of this service since July 1915.

We must further point out the following facts :

1. The average stay in hospital of 28 days might be further decreased, for the Convalescent Leave Commission only sits once a week, on Thursdays, and were it not for this, some of the patients who become fit to leave between two meetings of the Commission, might take their departure several days earlier.

2. Each month the service receives surgical units which work in it for a certain time in order to become acquainted with the method used. These surgeons have a prudent tendency at first, naturally enough, to practice delayed primary and secondary suture rather than primary, which, of course, increases the length of stay in hospital.

3. The service receives, in principle, all wounds of soft parts which are sent to an evacuation center. This includes, of course, many serious wounds of the large vessels, the nerves, the articulations and numerous bone wounds — in some cases, even, complete fractures.

*Chart showing Primary, Delayed Primary, and Secondary Sutures (successes, partial successes and failures) during two periods.*

REPORT BY DR. LEMAITRE

*First Period. — All wounds.*

July 1915 to July 1917

| | | |
|---|---|---|
| Early primary sutures . . . . . . . . | 1046 | 13 failures<br>39 partial successes |
| Complete successes . . . . . . . . . . | | 994 |
| Delayed primary sutures . . . . . . . | 115 | 8 failures<br>9 partial successes |
| Complete successes . . . . . . . . . . | | 98 |
| Secondary sutures . . . . . . . . . . | 191 | 9 failures<br>13 partial successes |
| Complete successes . . . . . . . . . . | | 170 |
| Wounds not sutured . . . . . . . . . | | 768 |

*Second Period. — Soft Parts.*

July 1917 to February 1st 1918.

| | | |
|---|---|---|
| Primary sutures. . . . . . . . . . . . | 1618 | 19 failures<br>44 partial successes |
| Complete successes. . . . . . . . . . | | 1555 |
| Delayed primary sutures. . . . . . . | 116 | 5 failures<br>7 partial successes |
| Complete successes. . . . . . . . . . | | 104 |
| Secondary sutures. . . . . . . . . . . | 133 | 5 failures<br>11 partial successes |
| Complete successes. . . . . . . . . . | | 117 |
| Wounds not sutured. . . . . . . . . | | 85 |

— Since the delivery of this lecture, the service of Doctor Lemaître has been transferred to the H. O. E. of Cravant, Yonne, where the conditions under which the patients are received are wholly different from those under which Doctor Lemaître had previously worked. Up to the time of his transfer, indeed, the proximity of the front had made it possible for the wounded to be brought to the operating table within a few hours, whereas, on the contrary, at Cravant it was impossible to operate before the thirtieth hour, and sometimes the wounded arrived as late as 36 or even 48 hours after the injury. It was especially interesting to note at the beginning of this new establishment that, in spite of the age of the wounds, they could, nevertheless, be treated by immediate primary suture in a very large proportion of the cases, without any more accidents than in the cases previously observed. No new statistics, accurately tabulated, have yet been collected and verified, but in general we may say that 60 o/o of the wounds which we have received at this station, after 2 or even 3 days from the time of the injury, have been sutured immediately with complete success. This percentage, which is excellent in itself, is of course lower than in the statistics of 1917 (80 o/o); but it should be observed at once that the percentage of wounds which were not sutured (or sutured only secondarily) has not increased for this reason : for the difference is more than made up by the cases closed by delayed primary suture, the percentage of which has jumped from 6 to 20 o/o. The age of the wound, which is one of the classic contra-indications for primary suture, is an indication for delayed primary suture.

It is this delayed primary suture which is to be considered in this supplementary chapter.

The indications are to be found in the three following conditions:

1. Age of the wound.

2. A slight degree of infection, noticeable clinically at the time of the examination preceding operation.

3. Military necessity, requiring the evacuation of the wounded before the expiration of the minimum time necessary for the surveillance of a wound primarily sutured.

In reality, the delayed suture may be practised in almost all cases. It is almost as good as the immediate primary suture is for wounds treated by this latter method. It has the advantage over them of absolute security, since it can be carried out only after the lapse of several days, when the surgeon can have noted *de visu* the good condition of the wound. When this method is followed, all risk of danger is avoided, and thus the criticism most frequently made against the method is disarmed. But the special interest of delayed primary suture for those who have the technic of preliminary ablation well in hand is that it permits the early closing of all wounds, the immediate closing of which does not appear advisable, but in which subsequent evolution is favorable.

Delayed primary suture may be practised in the case of:

1. Wounds which might be closed by immediate primary suture when the operator has had no experience in this method.

2. Wounds which have not been closed by immediate suture for reasons of prudence, but which at the first and second dressing appear to be in a condition for suturing.

3. Wounds which are not sutured immediately because the patient is in a state of shock, or suffering from multiple wounds.

4. Wounds which must be operated several days after the injury.

5. Wounds which present a slight degree of infection at the time of the operation (edema, lymphangitis), but upon which careful ablation has produced quick results.

6. Wounds which, at the first dressing, present scabs which are eliminated at the second or third dressing.

7. Wounds, slightly infected, but sterilized for several days by auto-vaccination, provided, of course, that granulation has not begun.

8. Wounds, the vitality of which, and consequently the power to resist infection, was doubtful because of a ligature immediately above the wound.

9. Wounds in which there is a minute bit of projectile which could not be removed at the time of the operation, but which show no ill effects due to its presence.

10. Wounds already operated and cleaned which were not sutured for reasons of military necessity, which required evacuation before the lapse of the period considered indispensable for the proper surveillance of sutured wounds.

The best moment for delayed primary suture is the fourth day after operation; this is the time at which, in the service of Dr. Lemaitre, we do the first dressing of wounds which have not been sutured, provided the patient is without pain or fever, and the bandage has not become soiled at an earlier date. If the wound is not sufficiently clean, it is best to delay suture for several days more; it may be practised as late as the twelfth day.

The operative technic is identical to that for immediate primary suture; rather than suturing the whole *en masse* with silkworm gut, it is better to suture layer by layer with fine catgut in order to re-establish the anatomical layers, and especially to make possible the natural gliding of the tissues. The only special point to be noted in practising the delayed technic, is the following: the surface of the resected derma is rapidly covered with an epidermic border which sometimes extends for several millimeters. It is necessary, before practising cutaneous suture, to separate with a grooved director this epidermic border (to resect it, if necessary because of its length) so as to bring the edges of the derma precisely together when sutured, thus making possible a good scar, which shall be linear, firm, and supple. As in immediate suture, the stitches may be removed after the tenth or twelfth day, and no change of dressing will be necessary before that time, provided the patient is without pain, a rapid pulse, or persistent fever. If a filiform drain has been inserted it may be removed on the fourth day.

For those who know how to effect a good ablation, and who have the technic of delayed primary suture well in hand, the possibilities of this procedure are infinite. Thus between the fourth and the twelfth day we have been able to close wounds with extensive gas gangrene, even when accompanied by fracture, in which cases the best of results have been obtained.

(Additional note by Dr. René Bloch, Chief Surgeon of Team A of Dr. Lemaitre's service.)

